# Identification and Expression Profiling of the Auxin Response Factors in *Capsicum annuum* L. under Abiotic Stress and Hormone Treatments

**DOI:** 10.3390/ijms18122719

**Published:** 2017-12-15

**Authors:** Chenliang Yu, Yihua Zhan, Xuping Feng, Zong-An Huang, Chendong Sun

**Affiliations:** 1 Vegetable Research Institute, Zhejiang Academy of Agricultural Sciences, Hangzhou 310021, China; 2 State Key Laboratory of Plant Physiology and Biochemistry, College of Life Sciences, Zhejiang University, Hangzhou 310058, China; 21107014@zju.edu.cn (Y.Z.); 11307001@zju.edu.cn (C.S.); 3 Key Laboratory of Spectroscopy, Ministry of Agriculture, College of Biosystems Engineering and Food Science, Zhejiang University, Hangzhou 310058, China; pimmmx@163.com; 4 Institute of Vegetable Sciences, Wenzhou Academy of Agricultural Sciences, Wenzhou 325014, China; huangzongan1978@163.com

**Keywords:** abiotic stresses, auxin, auxin response factor, *Capsicum annuum*, expression pattern

## Abstract

Auxin response factors (ARFs) play important roles in regulating plant growth and development and response to environmental stress. An exhaustive analysis of the *CaARF* family was performed using the latest publicly available genome for pepper (*Capsicum annuum* L.). In total, 22 non-redundant *CaARF* gene family members in six classes were analyzed, including chromosome locations, gene structures, conserved motifs of proteins, phylogenetic relationships and Subcellular localization. Phylogenetic analysis of the ARFs from pepper (*Capsicum annuum* L.), tomato (*Solanum lycopersicum* L.), *Arabidopsis* and rice (*Oryza sativa* L.) revealed both similarity and divergence between the four ARF families, and aided in predicting biological functions of the CaARFs. Furthermore, expression profiling of *CaARF*s was obtained in various organs and tissues using quantitative real-time RT-PCR (qRT-PCR). Expression analysis of these genes was also conducted with various hormones and abiotic treatments using qRT-PCR. Most *CaARF* genes were regulated by exogenous hormone treatments at the transcriptional level, and many *CaARF* genes were altered by abiotic stress. Systematic analysis of *CaARF* genes is imperative to elucidate the roles of *CaARF* family members in mediating auxin signaling in the adaptation of pepper to a challenging environment.

## 1. Introduction

Auxin plays crucial roles in several aspects of plant growth and developmental processes, including embryogenesis, vascular elongation, apical dominance, root architecture, flower and fruit development, organ initiation and patterning, secondary metabolism, and responses to environmental stimuli [1,2,3]. Auxin response factors (ARFs) and auxin/indole-3-acetic acid (Aux/IAA) are important transcription factor families that can modulate/regulate the expression of auxin early-responsive gene families including *Small Auxin Up RNA* (*SAUR*) and *Gretchen Hagen3* (*GH3*) gene families [4]. ARFs regulate the expression levels of auxin response genes by binding to the promoters of auxin response elements (AuxREs): TGTCTC or some variant of this element (e.g., TGTCCC or TGTCAC) [5,6]. 

Most ARFs consists of three conserved domains: a B3 DNA binding domain (DBD) which can recognize AuxREs in the promoter of auxin-responsive genes in N-terminal; a variable middle region (MR) that may function as an activation or repression domain; and a conserved Carboxy-Terminal Domains (CTD), which is participated in protein interaction with Aux/IAA proteins [5,7]. In recent years, several mutants of *ARF* genes have been screened to study their genetic functions. For example, the leaf senescence of the *atarf2* mutant is delayed in *Arabidopsis* [8]. The genes *AtARF2*, *7* and *19* were induced by senescence, and mutations in *AtARF7* and *19* enhanced *atarf2* phenotypes [9]. *Atarf3* mutation caused deviant floral meristem and reproductive organs [10]. *ETTIN* (*ETT*/*AtARF3*) and *AtARF4* control the development of floral organs and leaves in *Arabidopsis* [11]. Abnormal formation of vascular bundle and embryonic axis are observed in the *atarf5* mutant [12,13]. *AtARF6* and *AtARF8* affect the timing of flower maturation [14]. *Atarf7* mutation blocks the hypocotyl response to blue light and reduces the auxin response [15]. *AtARF7*/*AtARF19* double mutant affect lateral root formation and have abnormal gravitropism in both hypocotyls and roots [16,17]. *AtARF10* targeted by *AtmicroRNA160* functions in the regulation of seed germination and post-germination [18,19]. In rice (*Oryza sativa*), *OsARF12* is involved in iron homeostasis [20]. Another *ARF*, *OsARF16*, plays a role in efficient utilization of iron and is also required for iron deficiency response [21,22]. *SlARF3* is involved in the formation of trichomes and epidermal cells in tomato [23]. *SlARF7* moderates the auxin response during fruit growth and acts as a negative regulator of fruit set until pollination and fertilization have taken place [24].

Auxin plays a vital role in the spatiotemporal coordination of plant tolerance to stress [25,26]. Auxin signaling is initiated via activation of transcriptional response by the ARF transcription factors. Since *AtARF1* was first cloned from *Arabidopsis*, 17 members in *Solanum lycopersicon*, 19 in *Citrus sinensis*, 25 in rice, 24 in *Medicago truncatula*, 31 in *Zea mays*, 39 in *Populus trichocarpa* and 35 in *Gossypium raimondii* have been identified [6,27,28,29,30,31,32]. However, little is known about the *ARF* family genes in pepper (*Capsicum annuum* L.). Pepper is an economically important agricultural *Solanaceous* vegetable and is susceptible to abiotic stresses, including salinity and extreme temperature. In this study, we provide comprehensive information on the 22 *CaARF* genes, and investigate their expression patterns after exposure to salt and extreme temperatures, as well as various hormone treatments. 

## 2. Results

### 2.1. Genome-Wide Identification of CaARF Genes from Pepper

**Figure 1:**
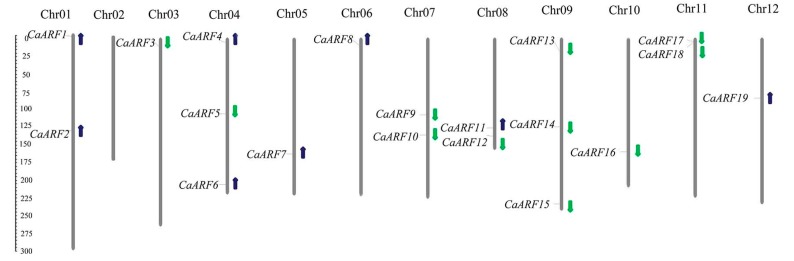
Chromosomal distribution analysis of *CaARF* family genes in pepper. MapInspect software was used to draw the location images of *CaARF* genes. Pepper chromosomes were arranged in blocks by its relative length. The chromosome number is indicated at the top of each chromosome. 19 *MtARF* genes were among eight chromosomes except chromosomes 2. The direction of transcriptions were indicated by arrows. The scale is megabases (Mb).

After an extensive BLAST search, 22 non-redundant *ARF* genes were identified in pepper. These *CaARF*s were named as *CaARF1*–*CaARF22* according to their locations on chromosomes (Table 1, Figure 1). Information on *CaARF* gene families such as gene names, gene IDs, open reading frame (ORF) lengths, number of exons, chromosomal localization, domains and parameters for the deduced polypeptides are listed in Table 1. The number of ARF genes in pepper is similar to that in *Arabidopsis* (23), rice (25) and *M. truncatula* (24), and more than the number in tomato (17). The ORF sizes for the *CaARF* genes varied from 621 bp (*CaARF14*) to 3384 bp (*CaARF9*), and the sizes of deduced CaARF proteins were varied 206 from 1125 amino acids. The predicted molecular weights of CaARF proteins ranged from 23,695.03 Da (CaARF14) to 124,451.92 Da (CaARF9). The predicted pI ranged from 5.190 (CaARF4) to 10.386 (CaARF6). Pair-wise analysis of CaARF proteins indicated that the overall identity fell in a range from 22.39% (between CaARF5 and CaARF22) to 90.65% (between CaARF6 and CaARF22) (Figure S1).

### 2.2. Chromosomal Distributions

Based on the start positions of the *CaARF* genes on chromosomes, we mapped all 22 genes onto the chromosomes (Table 1 and Figure 1). There were 19 *CaARF* genes unevenly mapped on 11 out of 12 pepper chromosomes, except for chromosome 2. Three unmapped *CaARF* genes were present on a pseudo-chromosome (chr00). Chromosomes 3, 5, 6, 10 and 12 contained only one *CaARF* gene; two genes were located on chromosomes 1, 7, 8 and 11; and three *CaARF* genes were distributed on chromosomes 4 and 9 (Table 1, Figure 1).

### 2.3. Phylogenetic Relationship Analyses and Gene Structure

**Figure 2:**
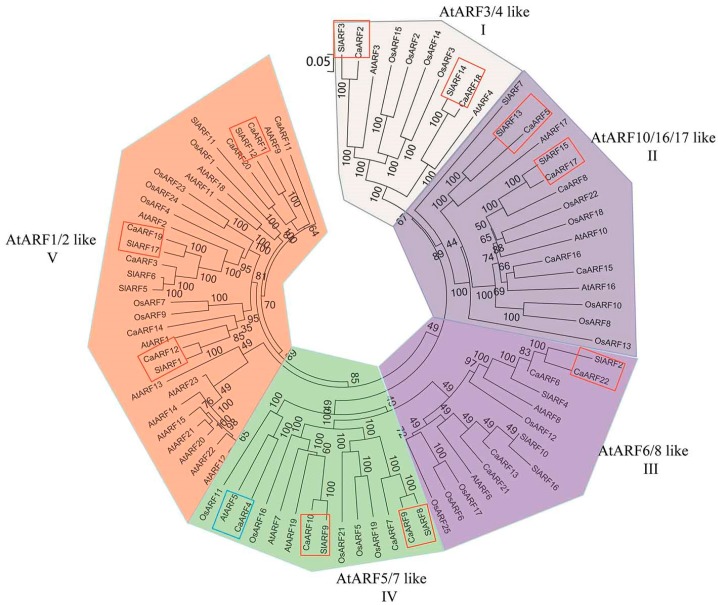
Phylogenetic relationship analysis of ARF family between Pepper, tomato, *Arabidopsis* and rice. All branches were marked with bootstrap values. The orthologous genes between pepper and tomato were indicated by red boxes. The orthologous genes between pepper and *Arabidopsis* were indicated by blue boxes.

The biological functions of several *ARF* genes have been reported in *Arabidopsis*, rice and tomato [8,14,15,20,24]. Investigations into the evolutionary relationships of ARF proteins among pepper, *Arabidopsis*, tomato and rice would booster our understanding of the possible biological functions of these genes. The amino acid alignments of the ARF family proteins from *Arabidopsis*, tomato, rice and pepper were performed using the Clustal Omega program. A phylogenetic tree comprising 87 *ARF* genes from *Arabidopsis* (23), tomato (17), rice (25) and pepper (22) was constructed using MEGA6.0 software with the neighbor-joining method (Figure 2). The phylogenetic distribution showed that the ARF family could be classified into five major classes: I (AtARF3/4-like), II (AtARF10/16/17-like), III (AtARF6/8/10-like), IV (AtARF5/7-like), and V (AtARF1/2-like). CaARF2 and CaARF18 from pepper were clustered in class I; 5 members (CaARF5, 8, 15, 16 and 17 in class II; CaARF6, 13, 21 and 22 in class III. CaARF4, 7, 9 and 10 in class IV; and CaARF1, 3, 11, 12, 14, 19 and 20 in class V. Based on the phylogenetic tree, 10 sister-gene pairs were identified between tomato and pepper: *CaARF1*/*SlARF12*, *CaARF2*/*SlARF3*, *CaARF5*/*SlARF13*, *CaARF9*/*SlARF8*, *CaARF10*/*SlARF9*, *CaARF18*/*SlARF14*, *CaARF12*/*SlARF1*, *CaARF19*/*SlARF17*, *CaARF17*/*SlARF15* and *CaARF22*/*SlARF2*. A sister gene pair was identified between *Arabidopsis* and pepper: *CaARF4*/*AtARF5*.

The intron-exon structures of *CaARF* genes were determined by comparison of cDNA sequences with genomic DNA sequences. This gene sequence structure revealed introns in all *CaARF* genes. There were 2 to 16 exons in *CaARF* genes (Figure S2).

### 2.4. Analysis of Protein Structure and Prediction of miRNA Targets among CaARFs

**Figure 3:**
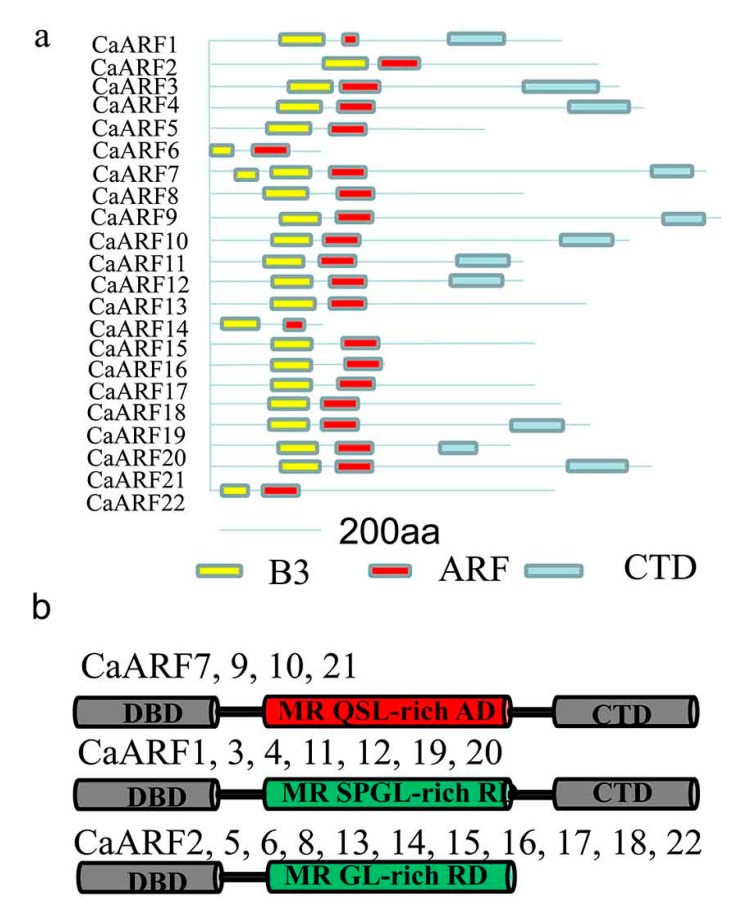
Analysis of ARF protein structures and domains. (**a**) Depiction of the domain structure of each CaARF protein sequence. The B3 DNA bindingdomain (BDB), auxin response factor domain (ARF) and Carboxy-Terminal Domains (CTD) are colored in yellow, red and light blue, respectively. (**b**) Three kinds of CaARF protein structures. DBD, DNA-binding domain; CTD, C-terminal dimerization domain; MR, middle region; RD, repression domain, showed in green color ; AD, activation domain, showed in red color; Q, glutamine; S, serine; L, leucine; P, proline; G, glycine.

The 22 CaARF proteins were divided into three groups, based on their variable middle region (MR) amino acid composition and the presence or absence of CTD: (1) ARF proteins with a B3 DNA binding domain (DBD), activator MR (MR enriched with glutamine (Q), serine (S), and leucine (L)) and a CTD; (2) ARF proteins with a DBD, repressor MR (MR enriched with S, L, proline (P) and glycine (G)) and a CTD; and (3) ARF proteins with a DBD and repressor MR, but no CTD (Figure 3). The *CaARF* family encodes four putative transcriptional activators: CaARF7, 9, 10 and 21; and seven putative transcriptional repressors: CaARF1, 3, 4, 11, 12, 19 and 20. Eleven CaARF proteins did not contain a CTD: CaARF2, 5, 6, 8, 13, 14, 15, 16, 17, 18 and 22 (Figure 3).

To further investigate conserved motifs, the Multiple Expectation Maximization for Motif Elicitation (MEME) software was employed to characterize the distribution of motifs in 22 CaARF proteins. There 15 motifs identified and mapped to the CaARF amino acid sequences (Figure S3). CaARF7 and CaARF9 contained the greatest number (14 motifs), and CaARF14 the fewest motifs (five motifs). Motifs 1, 2 and 7 corresponded to the B3-DBDs; motifs5, 8, 12 and 15 corresponded to ARF domain; and motifs 4 and 10 corresponded to the OPCA-like motif((D-x-D/E-x-D-xn-D/E) known as the octicosapeptide repeat, p40phox and budding yeast Cdc24p, a typical PKC interaction domain (OPCA) motif) and conserved lysine motif of the PB1 domain, which function in protein-protein interaction with Aux/IAA [33,34].

Previous studies showed that expression levels of several *ARF* genes were regulated by miRNA in *Arabidopsis*. For example, *AtARF6* and *8* were targeted by *AtmiRNA167* [35,36,37,38]; and *AtARF10*, *16*, and *17* regulated by *AtmiRNA160* [18,19]. To explore whether there was potential regulation of *CaARF* genes by miRNA, putative target sites were searched using the BLASTN algorithm. Target sites of *AtmiRNA160* (UGCCUGGCUCCCUGGAUGCCA) were predicted within the mRNA region of *CaARF5*, *8*, *15* and *17* (Figure S4a). Additionally, target sites of *AtmiRNA 167* (UCAAGCUGCCUGCAUGAUCUA) were predicted within the mRNA region of *CaARF13*, *21* and *22* (Figure S4b). The phylogenetic tree placed *CaARF13*, *21* and *22*, *AtARF6* and *8* in class III; and *CaARF5*, *8*, *15*, and *17*, *AtARF10*, *16* and *17* in class II. The results of miRNA targets analysis suggested that miRNA160-/167-mediated post-transcriptional regulation of ARFs was conserved between pepper and *Arabidopsis*.

### 2.5. Analyses of Tissue-Specific Expressions and Subcellular Location

**Figure 4:**
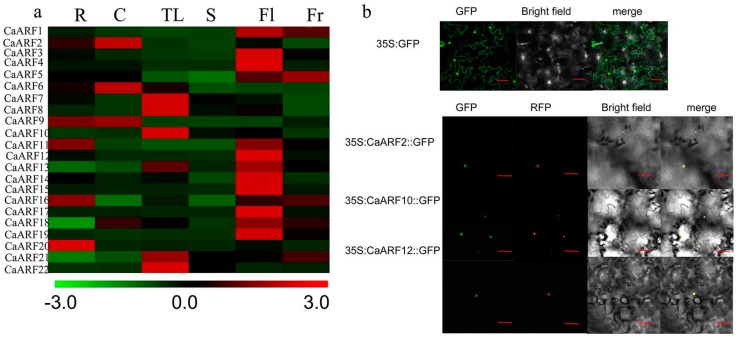
Tissues-specific expressions analysis of *CaARF* genes and Subcellular localization of three selected CaARF proteins. (**a**) Expression patterns of *CaARF* genes in six tissues. R: roots; C: cotyledons; TL: true leaves; S: stems; Fl: flowers; Fr: fruits. Levels of different colours were shown on expression scale of each *CaARF* genes. The value of *CaACTIN* defines as 1000 and the relative mRNA level of individual genes was normalized with respect to the *CaACTIN* gene. Heat map was draw by MeV software was using the average log_10_ (expression value) (**b**) *CaARF*s and GFP fusion proteins were transiently expressed in tobacco epidermis cells under the CaMV35S promoter. From top to bottom: 35S:GFP, 35S:CaARF2::GFP, 35S:CaARF10::GFP, 35S:CaARF12::GFP. Left to right: GFP fluorescence, red fluorescence of nuclear marker (NSL-mCherry), bright-field, merged microscope images. Bar = 50 μm.

To probe the biological function of *CaARF* genes, quantitative real-time RT-PCR (qRT-PCR) was used to determine the spatial specificity expression pattern of 22 *CaARF* genes in six pepper organs: roots, cotyledons, true leaves, stems, flowers and fruits) (Figure 4a). Transcripts of the 22 *CaARF* genes were detected in different organs and tissues, indicating multiple functions of *CaARF* genes in pepper growth and development. Some *CaARF* genes exhibited organ/tissue-specific expression patterns. *CaARF20* was especially expressed in fruits. *CaARF2* and *CaARF6* were highly expressed in cotyledons. *CaARF7*, *8*, *10*, *21* and *22* were expressed more strongly in true leaves than in the other tissues/organs. *CaARF1*, *3*, *4*, *12*, *14*, *15*, *17*, *18* and *19* were highly expressed in flowers compared with other tissues.

According to previous reports, most ARF proteins were nuclear-localized. Based on the amino acid composition and conserved motifs analysis, CaARF2, CaARF10 and CaARF12 from three different groups were fused in-frame to the N-terminus of the green fluorescence protein (GFP). The subcellular location of CaARF2, CaARF10 and CaARF12 in the epidermis cells of tobacco leaves showed that these proteins were nuclear-localized (Figure 4b).

### 2.6. Expression Patterns of CaARF Genes in Response to Various Abiotic Stress Treatments

**Figure 5:**
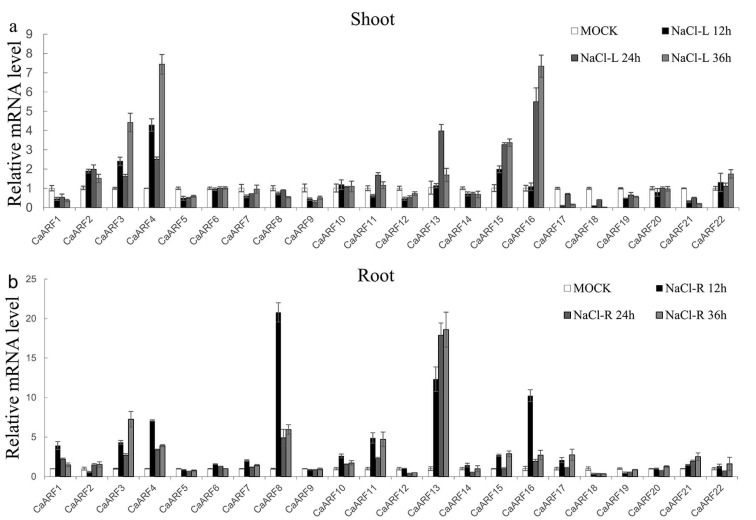
Expression profiles analysis of *CaARF* family genes in response to salt stress in both shoots (**a**) and roots (**b**). Expression levels of *CaARF* family genes were analysed by qRT-PCR using 4-week-old pepper seedlings, which were treated with 200 mM NaCl for 36 h. The relative expression levels were normalized to a value of 1 in mock seedlings. Standard deviations were shown with error bars.

**Figure 6:**
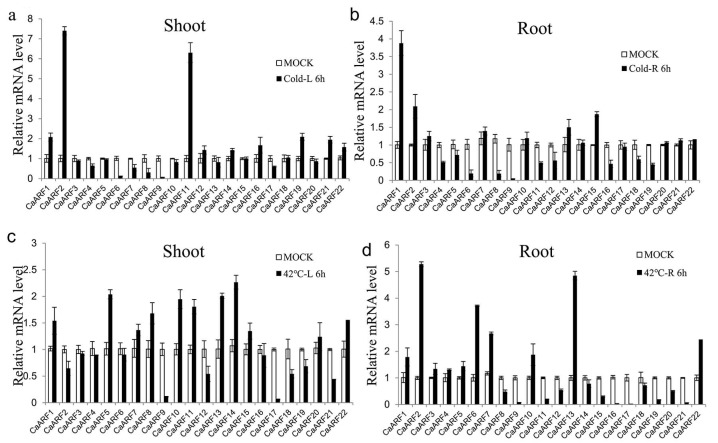
Expression profiles analysis of *CaARF* family genes in response to cold and heat stress. The expression levels of *CaARF* genes in cold (**a**) and heat (**c**) treated shoots were compared to mock shoots as relative mRNA levels. The expression levels of *CaARF* genes in cold (**b**) and heat (**d**) treated shoots were compared to mock shoots as relative mRNA levels. Expression levels of *CaARF* family genes were analysed by qRT-PCR using 4-week-old pepper seedlings, which were grown under 4 or 42 °C for 6 h, respectively. The relative expression levels were normalized to a value of 1 in mock seedlings. Standard deviations were shown with error bars.

Pepper is sensitive to stress factors, such as extreme temperature, salinity and osmotic stresses [39,40]. Many studies have shown that auxin and its signaling are involved in stress responses, including to drought, cold and salt [36]. In this study, expression levels of *CaARF* genes under NaCl, cold (4 °C) and heat stress (42 °C) treatments were determined by qRT-PCR to investigate their potential roles in pepper responses to various abiotic stresses. Under these stresses, the majority of *CaARF* genes exhibited different expression patterns. Under the NaCl treatment, 10 (45.45%) *CaARF* genes were down-regulated in shoots and nine (40.9%) were up-regulated in roots (Figure 5). Following the 4 °C treatment, the expression levels of *CaARF4*, *6*, *8* and *17* were declined in both shoots and roots; *CaARF1* and *2* were up-regulated in both shoots and roots; and *CaARF11* was up-regulated in shoots and down-regulated in roots (Figure 6a). *CaARF2*, *9*, *12*, *17*, *18*, *19* and *21* were down-regulated in shoots after high temperature (42 °C) treatment. *CaARF2*, *6*, *7*, *13* and *22* were induced (value > 2) in roots after 42 °C treatment (Figure 6b).

### 2.7. Expression of CaARF Genes in Response to Various Hormone Treatments

**Figure 7:**
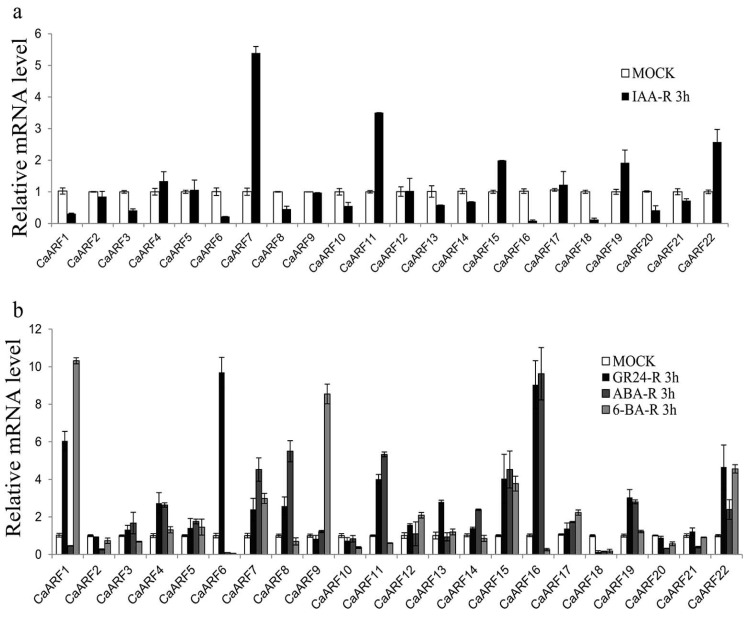
qRT-PCR analysis of *CaARF* family genes in roots under various hormone treatments. (**a**) Expression patterns of *CaARF* family genes family genes under 10 μM IAA treatment for 3 h. (**b**) Expression profiles analysis of *CaARF* family genes under 10 μM GR24, 1 μM ABA, 1 μM 6-BA for 3 h, respectively. Error bars represent standard deviations from 3 biological replicates.

The expression patterns of *CaARF* genes in response to exogenous IAA stimulation were analyzed using qRT-PCR. The expression levels of *CaARF 11*, *15*, *19* and *CaARF22* were induced by IAA treatment (Figure 7a). To investigate whether the *CaARF* gene family was involved in other hormone signaling pathways, we also analyzed expression profiles of these genes under other various hormone treatments. The transcriptional expression of the *CaARF* family genes in pepper seedlings were respectively tested using qRT-PCR following treatments with a synthetic analogue of strigolactone (GR24), abscisic acid (ABA) and 6-benzylaminopurine (6-BA) treatments. Following the GR24 treatment, expression levels of *CaARF4*, 6, *7*, *8*, *11*, *13*, *15*, *16*, *19* and *22* were up-regulated (>2 fold) (Figure 7b). The ABA treatment resulted in down-regulation of *CaARF1*, *2*, *6*, *10*, *18*, *20* and *21*. The 6-BA treatment induced up-regulation of *CaARF1*, *7*, *9*, *12*, *15*, *17* and *22* (>2 fold) (Figure 7b). Our results suggested that expression levels of some *CaARF* genes were responsive to these hormones.

## 3. Discussion

The phytohormone auxin is involved in regulating organogenesis and patterning processes during plant growth and development [37]. The auxin signaling pathway is mainly composed of two types of transcription factors: ARF and Aux/IAAs [4,38]. The ARFs directly bind to AuxREs in the promoters of down-stream target genes and regulate their expression during development [41]. Genome-wide characterization and analysis of *CaARF*s would help determine their mechanisms in pepper growth and development. We identified 22 *ARF* genes in pepper, a similar number to that of *Arabidopsis* (23). Domain analysis of CaARF proteins provided useful information for predicting their biological functions in pepper. ARFs binding to AuxRE (TGTCTC) in the promoters of auxin-responsive genes rely on the DBD domain. The ARF transcription factors function as activators or repressors, determined by the amino acid composition of MR [42]. The ratio of activator/repressor among ARF proteins in pepper was only 0.22 less than half of that in *Arabidopsis* (0.59) or rice (0.56) [43]. The CTD/PB1 domain of ARF protein is involved in resembling domains III and IV of Aux/IAA proteins [44]. The ratio of CTD-truncated CaARFs (50%) was much higher than that in soybean (15.68%), *Arabidopsis* (17.39%), *Brassica rapa* (22.58%) and rice (24%) [45,46]. These results suggested that some *ARF* genes in pepper might be regulated in an auxin independent way. Recently, similar work has been published by Zhang et al. [47]. The reason of the differences about the number (19 *CaARF* genes) and domain structure of the CaARF proteins obtained by Zhang et al. (2017) might be that different databases were used in this study [47]. Zhang et al. (2017) used the pepper genome database (https://solgenomics.net/organism/Capsicum_annuum/genome), SMART, Motif Scan (http://myhits.isb-sib.ch/cgi-bin/PFSCAN) and the MEME web server, while the Pepper Genomics Database 2.0, SMART and MEME were used in our study. 

A phylogenetic tree was constructed to analyse the relationship of ARF members between pepper, tomato, rice and *Arabidopsis* (Figure 2). The phylogenetic tree not only helped elucidate the phylogenetic relationships of ARF proteins, but also allowed speculation on putative functions of the CaARF proteins based on the functional clades previously described in tomato, rice and *Arabidopsis*. All of the *CaARF* genes were distributed in five classes, I–V, which were homologous to *AtARF3* and *4*; *AtARF10*, *16* and *17*; *AtARF6* and *8*; *AtARF5* and *7*; and *AtARF1* and *2*, respectively (Figure 2). The phylogenetic tree also showed 10 sister-gene pairs with high bootstrap values (≥99%) between pepper and tomato. The phylogenetic relationships of these pepper and tomato genes suggested the putative biological functions of the identified CaARFs [29,30]. Analysis of the protein motifs showed that different classes of CaARFs had a conserved structure. Motif 4 and 10, located in the PB1 domain, included a conserved lysine motif and an OPCA-like motif found only in the ARF family, indicating the evolutionary conservation of ARF function [33,48]. All 22 CaARF proteins have B3 and ARF domains that were predicted by SMART (Figure 3a). Many studies have shown that miRNA plays an important role in post-transcriptional gene regulation by combining with complementary targets. The phylogenetic tree showed that *CaARF5*, *8*, *15* and *17*, *AtARF10*, *16*, and *17* were in class II; and these four *CaARF* genes contained a target site for miRNA160. Additionally, *CaARF13*, *21* and *22*, *AtARF6* and 8 were clustered in class III, were a target for miRNA167.

Comprehensive expression patterns analysis in different organs/tissues using qRT-PCR helped us screen for *CaARF* genes with potentially distinct functions (Figure 4). Our data showed that most *CaARF* genes were highly variably expressed in all six organs/tissues. Their expression patterns suggest that the encoded proteins may perform diverse functions. *ARF* genes have previously been shown to be involved in regulating plant growth and development [8,15]. AtARF4 was reported to be involved in flower patterning [49]. In pepper, CaARF18 expression was significantly higher in flowers than in other studied organs, and was closely related to *AtARF4* in class I; these findings indicate that CaARF18 might play a crucial role in flower development. In *Arabidopsis*, auxin induces lateral roots and leaf expansion by activating ARF7 and *19* in *Arabidopsis* [50]. In pepper, *CaARF10* belonged to class IV with *AtARF7* and *19*, and *CaARF10* expression was much higher in pepper leaves than in other organs. These observations suggest that *CaARF10* likely regulates auxin-induced leaf expansion. A clear increase in transcriptional level of most *CaARF* genes was observed in adventitious root growth [47].

Phytohormones are involved in the responses of various plants to environmental stimuli and stress by altering the expression levels of many *ARF* genes [6,51]. However, evaluation of pepper’s adaptability to its environment is very limited. In this study, an expression profile of the *CaARF* family genes in response to various abiotic stresses and several hormones was created. Pepper is considered to be extremely sensitive to salt, cold and high temperature. However, few studies have examined the responses of pepper to abiotic stresses and related signal transduction. Genome-wide expression analysis showed that the transcriptional level of many *ARF* genes changes when plants response to abiotic stresses [52,53,54]. Auxin plays a key role in plant responses to abiotic stresses through complex metabolic and signaling networks [55]. In banana, many *MaARF* genes at the transcriptional level can respond to abiotic stresses [56]. More than half of the *GmARF* genes are dehydration responsive have been reported in soybean [54]. In this study, we found that many *CaARF* genes can respond to abiotic stresses including salt, cold and heat at the transcriptional level. Our data indicated essential roles of these genes in response to abiotic stresses in pepper, providing many excellent candidate genes for further studies.

Since ARFs regulate the expression of auxin response genes, it would be interesting to determine the response of *CaARF* genes to exogenous IAA treatment. It has been reported that *AtARF4*, *5*, *16*, *19* and *OsARF1* and *23* slightly increased under auxin treatment, whereas *OsARF5*, *14* and *21* decreased slightly [27,28,57]. In our study, expression of *CaARF* genes changed rapidly under exogenous auxin treatment compared with the control. In pepper, 12 of the 22 *CaARF* genes were down-regulated by IAA treatment in the roots. In addition to auxin, crosstalk between hormone and abiotic stress signaling has been reported in some plant species [52,58]. Strigolactones, a group of plant hormones, were recently described to be involved in the repression of shoot branching. Their signaling is required for auxin-dependent stimulation of secondary growth in plants [59]. In *Arabidopsis*, auxin acts upstream of *ABSCISICACID INSENSITIVE 3* (*ABI3*) by recruiting *AtARF10* and *16* to control expression of *ABI3* during seed germination [60]. Auxin and cytokinin play an antagonistic role in plant development; however, their interaction is much more complicated, depending on dose and cell type. Recent studies have begun to elucidate the molecular mechanisms involved in auxin–cytokinin interaction in biosynthesis, inactivation/degradation, transport and signal transduction [61,62]. In soybean, miRNA160 promotes auxin activity by suppressing the levels of the ARF10/16/17 family of repressor ARF transcription factors during nodule development. High miR160 levels promote auxin activity and suppress cytokinin activity, but low miR160 levels did the opposite [63].

## 4. Materials and Methods

### 4.1. Plant Material and Treatments

Pepper (*Capsicum annuum* L.) seeds, sown in perlite beds after sterilized by 1% sodium hypochlorite for 30 min. The seedlings were grown in a greenhouse under the following conditions: 16 h light (600 μE m^2^ s^−1^) at 26 °C, 8 h dark at 18 °C, and the relative humidity was 60%. Seedlings were irrigated by half-strength of Hoagland solution (pH 5.6). Seedlings at the four-true leaf stages were used for treatments. For salt treatment, plants were soaked in half-strong Hoagland nutrient solution containing 200 mM NaCl. For cold and hot treatments, seedlings were subjected to 4 °C or 42 °C Light incubator, respectively. For hormone treatments, plants were soaked in nutrient solution containing indole-3-acetic acid (IAA, 10 μM), *rac*-GR24 (10 μM), abscisic acid (ABA, 1 μM), or 6-Benzylaminopurine (6-BA, 1 μM), respectively. Plants grown in normal half-strong Hoagland nutrient solution were used as control. The root and shoot samples of pepper plants were harvested for RNA extraction. For tissue-specific expression analysis, roots, cotyledons, true leaves and stems samples were collected from two true leaf stages; for flower samples, flowers were collected after opening; for fruits samples, fruits were harvested at 15 days after anthesis. All experiments were repeated for 3 times. 

### 4.2. Identification of ARF Family Genes in Pepper

The sequences of *CaARF* were collected by homology screening against the Pepper Genomics Database 2.0 (http://peppersequence.genomics.cn/page/species/index.jsp). The known sequences of *Arabidopsis ARF*s (*AtARF*s) and tomato *ARF*s (*SlARF*) were used as queries. Information on *AtARF*s and *SlARF* was downloaded from the TAIR database (The Arabidopsis Information Resource, http://www.arabidopsis.org/) and the SGN database (The tomato Information Resource, http://solgenomics.net), respectively. Then, candidate genes were identified based on the hidden Markov model (HMM) profiles of the ARF gene family (Pfam 02362:B3 DNA binding domain (B3-DBD); Pfam 02309: AUX/IAA family (PB1); Pfam 06507:ARF (AUX_RESP)). To exclude redundant genes, all candidate *CaARF* genes were checked manually. All the obtained sequences are identified as unique sequences for further studies. The DNAstar tool (http://www.dnastar.com/) was employed to predict protein molecular weight (Mw) and isoelectric point (pI) of each *CaARF* proteins. 

### 4.3. Sequence Analysis, Genome Distribution, Phylogenetic Tree Building and Gene Structures

B3-DBD, ARF and BP1 domains were analyzed using SMART (http://smart.embl-heidelberg.de/) and the Multiple Expectation Maximization for Motif Elicitation (MEME) web server (http://meme.nbcr.net/meme/cgi-bin/meme.cgi). Optimum motif width was set from 6 to 200, and the maximum number of motifs was set to 15. The chromosomal location data of *CaARF* family genes were obtained from Pepper Genomics Database. A map of the distribution of *CaARF* family genes was drawn by MapInspect software (http://www.softsea.com/review/MapInspect.html) based on the chromosomal position of each *CaARF* gene.

The multiple sequence alignment file of ARF proteins from pepper, tomato, Arabidopsis and rice was generated by ClustalW program with the default parameters. Information on SlARF, AtARF and OsARF proteins was according to previous reports [6,27,32]. A neighbour-joining tree was constructed by MEGA6.0 (http://www.megasoftware.net/) with the p-distance and complete deletion parameters. Bootstrap analysis was calculated from 1000 iterations. Exon-intron structures of *CaARF* family genes were employed by Gene Structure Display Server (GSDS) tool (http://gsds.cbi.pku.edu.cn/) according to the full-length coding sequence and genome from Pepper Genomics Database. 

### 4.4. Subcellular Localization

The ORFs of *CaARF2*, *10* and *12* were cloned from pepper cDNA and integrated into the pEASY T1 vector (code: CT101-01, Transgen, Beijing, China). Then they were ultimately digested with *Kpn* I and *Sal* I, and ligated into the pCAMBIA1301 vector which contains a GEP gene. 35S: All fusion vectors were transiently expressed in epidermal cells of *N. benthamiana* leaves by agrobacterium-mediated transformation, respectively. NSL-mCherry was used as a nuclear marker. Micrographs were taken using two-photon microscopy LSM710 scanning system (Carl Zeiss, Oberkochen, Germany).

### 4.5. RNA Isolation and Quantitative Real Time-Polymerase Chain Reaction (qRT-PCR)

Total RNA was extracted from 0.05 g of samples using RNAprep pure Plant Kit (code: DP432, Tiangen, Beijing, China) according to the manufacturer’s instructions. DNase I was used to remove DNA contamination. The same amount of total RNA (1 μg) was used in each assay. Reverse transcription was performed using PrimeScript™ RT reagent Kit (code: RR047, TaKaRa, Kyoto, Japan) according to the manufacturer’s protocol. QRT-PCR was performed on LightCycler480 instrument (Roche, Basel, Switzerland) using SYBR^®^ Premix Ex Taq™ (Tli RNaseH Plus) (code: RR420, TaKaRa, Japan) with the primers listed in Table S1. The *CaACTIN* (*Capana12g001934*) was used as an internal standard. The relative expression levels were calculated using the 2^−ΔΔ*C*t^ method [64]. MeV software was employed to draw a heat map using the average log_10_ (expression value) to visualize the tissues-specific expression data. All the expression analyses were carried out with three biological repeats.

## 5. Conclusions

In conclusion, 22 *ARF* gene members in pepper were identified, and comprehensive information was collected. The conserved domains, phylogenetic relationship, the amino acid compositions, the gene structures, subcellular localizations and miRNA targets of *CaARF*s were analyzed in detail. The analysis of the expression patterns of *CaARF* genes in different tissues and organs will enable us to study the expression of those *ARF* genes in specific regions or in a limited time regulated manner. The responsiveness of the *CaARF* genes to various stresses and hormones suggests that CaARFs are involved in the pepper seedlings’ tolerance to abiotic stresses.

## Figures and Tables

**Table 1:** Characteristics of *CaARF* genes in pepper (*Capsicum annuum*).

Gene	Locus ID	ORF Length(bp)	No. of Exons	Chromosome	Domains	Deduced Polypeptid		
				No.		Length(aa)	Mw(Da)	pI
*CaARF1*	Capana01g000109	1869	13	1	DBD, ARF, PB1	622	69,361.85	6.608
*CaARF2*	Capana02g001847	2487	11	2	DBD, ARF	828	90,404.2	7.4
*CaARF3*	Capana03g000640	2535	14	3	DBD, ARF, PB1	844	94,051.49	6.528
*CaARF4*	Capana04g000259	2808	9	4	DBD, ARF, PB1	935	102,999.67	5.19
*CaARF5*	Capana04g001778	1749	2	4	DBD, ARF	582	64,136.74	6.707
*CaARF6*	Capana04g002497	660	6	4	DBD, ARF	219	24,895.03	10.386
*CaARF7*	Capana05g001658	3288	13	5	DBD, ARF, PB1	1095	120,930.6	6.207
*CaARF8*	Capana06g000514	2055	4	6	DBD, ARF	684	76,040.43	5.988
*CaARF9*	Capana07g000865	3384	15	7	DBD, ARF, PB1	1127	124,451.92	6.417
*CaARF10*	Capana07g000989	2745	16	7	DBD, ARF, PB1	914	102,060.74	5.772
*CaARF11*	Capana08g001056	2049	13	8	DBD, ARF, PB1	682	76,246.12	6.411
*CaARF12*	Capana08g001774	1980	14	8	DBD, ARF, PB1	659	73,752.73	6.273
*CaARF13*	Capana09g000452	2463	16	9	DBD, ARF	820	90,274.66	5.938
*CaARF14*	Capana09g001199	621	7	9	DBD, ARF	206	23,695.03	8.465
*CaARF15*	Capana09g002164	2082	3	9	DBD, ARF	693	76,014.18	6.753
*CaARF16*	Capana10g001461	1134	2	10	DBD, ARF	377	42,514.83	7.345
*CaARF17*	Capana11g000076	2067	4	11	DBD, ARF	688	76,060.18	8.296
*CaARF18*	Capana11g000105	2259	12	11	DBD, ARF	752	83,911.87	6.353
*CaARF19*	Capana12g001354	2499	14	12	DBD, ARF, PB1	832	92,780.98	6.568
*CaARF20*	Capana00g000236	1833	7	0	DBD, ARF	610	68,804.93	6.586
*CaARF21*	Capana00g001906	2676	14	0	DBD, ARF, PB1	891	98,644.16	6.208
*CaARF22*	Capana00g004127	1986	9	0	DBD, ARF	661	74,277.79	6.294

## Supplementary Materials

Supplementary materials can be found at www.mdpi.com/1422-0067/18/12/2719/s1.

## Abbreviations

ARF	Auxin response factor
Aux/IAA	Auxin/indole-3-acetic acid
GH3	Gretchen Hagen3
SAUR	Small Auxin Up RNA
AuxREs	auxin response elements
DBD	DNA-binding domain
AD	Activation domain
RD	Repression domain
CTD	C-terminal dimerization domain
ABA	Abscisic acid
6-BA	6-benzylaminopurine
HMM	Hidden Markov model
MEME	Multiple Expectation Maximization for Motif Elicitation
GFP	Green fluorescent protein
ORF	Open reading frame
