# Supplementary material for: Identification and Expression Profiling of the Auxin Response Factors in Capsicum annuum L. under Abiotic Stress and Hormone Treatments
Source: Int J Mol Sci. 2017 Dec 15;18(12):2719. doi: 10.3390/ijms18122719 (PMC5751320; doi:10.3390/ijms18122719)
Supplement: Supplementary file 1 [file ijms-18-02719-s001.pdf]

Supplementary

Identification and Expression Profiling of the Auxin Response Factors in *Capsicum annuum* L. under Abiotic Stress and Hormone Treatments

Chenliang Yu [1,*], Yihua Zhan [2], Xuping Feng [3], Zong-An Huang [4] and Chendong Sun [2]

1. Vegetable Research Institute, Zhejiang Academy of Agricultural Sciences, Hangzhou 310021, China
2. State Key Laboratory of Plant Physiology and Biochemistry, College of Life Sciences, Zhejiang University, Hangzhou 310058, China; 21107014@zju.edu.cn (Y.Z.); 100550991@qq.com (C.S.)
3. Key Laboratory of Spectroscopy, Ministry of Agriculture, College of Biosystems Engineering and Food Science, Zhejiang University, Hangzhou 310058, China; pimmmx@163.com
4. Institute of Vegetable Sciences, Wenzhou Academy of Agricultural Sciences, Wenzhou 325014, China; huangzongan1978@163.com

* Correspondence: 21007030@zju.edu.cn; Tel.: +86-18768141629

1: CaARF5	100.00																					
2: CaARF15	40.58	100.00																				
3: CaARF16	47.65	73.42	100.00																			
4: CaARF8	40.78	60.37	59.66	100.00																		
5: CaARF17	42.88	64.98	64.47	69.45	100.00																	
6: CaARF18	30.13	32.51	39.09	31.36	33.96	100.00																
7: CaARF7	26.98	32.96	38.94	31.40	32.79	37.37	100.00															
8: CaARF9	27.53	34.02	40.99	33.66	33.82	38.57	79.26	100.00														
9: CaARF4	31.39	31.66	40.45	30.47	31.82	39.91	44.50	45.07	100.00													
10: CaARF10	29.65	33.86	41.48	33.04	34.15	37.48	53.62	56.29	47.71	100.00												
11: CaARF21	28.48	36.30	43.35	32.99	34.34	37.52	45.45	46.00	46.46	49.44	100.00											
12: CaARF13	28.57	35.64	42.72	33.87	35.24	39.20	46.38	47.73	48.75	51.47	79.31	100.00										
13: CaARF6	29.90	39.90	40.99	34.98	39.32	44.17	67.45	69.01	63.03	68.10	85.12	83.03	100.00									
14: CaARF22	22.39	29.69	41.03	27.85	29.22	29.21	40.68	41.03	40.73	42.37	52.35	54.64	90.65	100.00								
15: CaARF2	31.19	32.27	39.81	32.32	32.80	42.88	31.27	31.92	31.34	32.48	33.39	34.49	38.28	26.41	100.00							
16: CaARF1	31.08	32.68	40.81	32.14	31.68	40.56	35.94	37.54	37.30	38.66	39.19	40.00	48.84	37.22	32.94	100.00						
17: CaARF11	31.93	32.54	40.92	30.76	31.12	40.36	37.18	39.21	38.23	39.21	41.25	39.83	48.80	35.05	35.00	54.93	100.00					
18: CaARF20	31.62	34.96	42.16	32.95	33.14	41.05	38.22	39.58	37.96	39.82	41.25	40.52	47.85	37.84	37.55	64.80	60.99	100.00				
19: CaARF3	29.41	31.95	42.26	31.73	31.19	40.76	36.03	36.81	36.60	37.27	39.32	40.83	48.34	33.73	35.85	47.47	49.85	51.25	100.00			
20: CaARF19	29.23	32.99	42.26	31.79	31.41	39.44	35.32	36.50	35.44	36.44	38.31	39.47	46.92	31.18	35.31	46.67	47.66	48.74	77.32	100.00		
21: CaARF12	33.41	36.00	41.75	34.35	33.52	43.93	39.31	40.62	38.72	40.77	41.54	41.00	50.71	35.41	36.94	51.73	54.84	54.84	52.93	50.30	100.00	
22: CaARF14	37.20	41.57	40.00	41.72	39.88	47.53	42.94	44.07	44.38	42.86	51.41	50.28	44.09	45.08	43.82	61.19	65.00	64.07	58.99	55.80	86.81	100.00

Figure S1. Sequence identity matrix of pepper ARF proteins. Clustal Omega (http://www.ebi.ac.uk/Tools/msa/clustalo/) program were employed to examine sequence identity of 22 pepper ARF proteins.

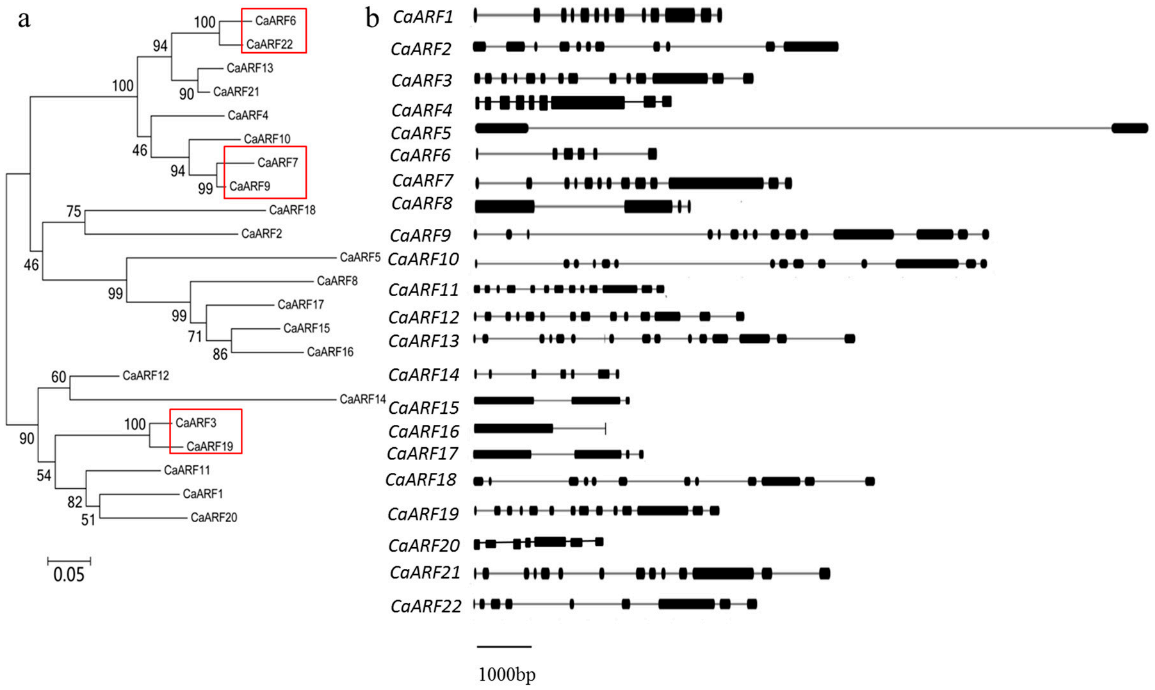

Figure S2: Phylogenetic relationships of CaARF proteins (a) and Gene structure analysis of *CaARF* genes (b).

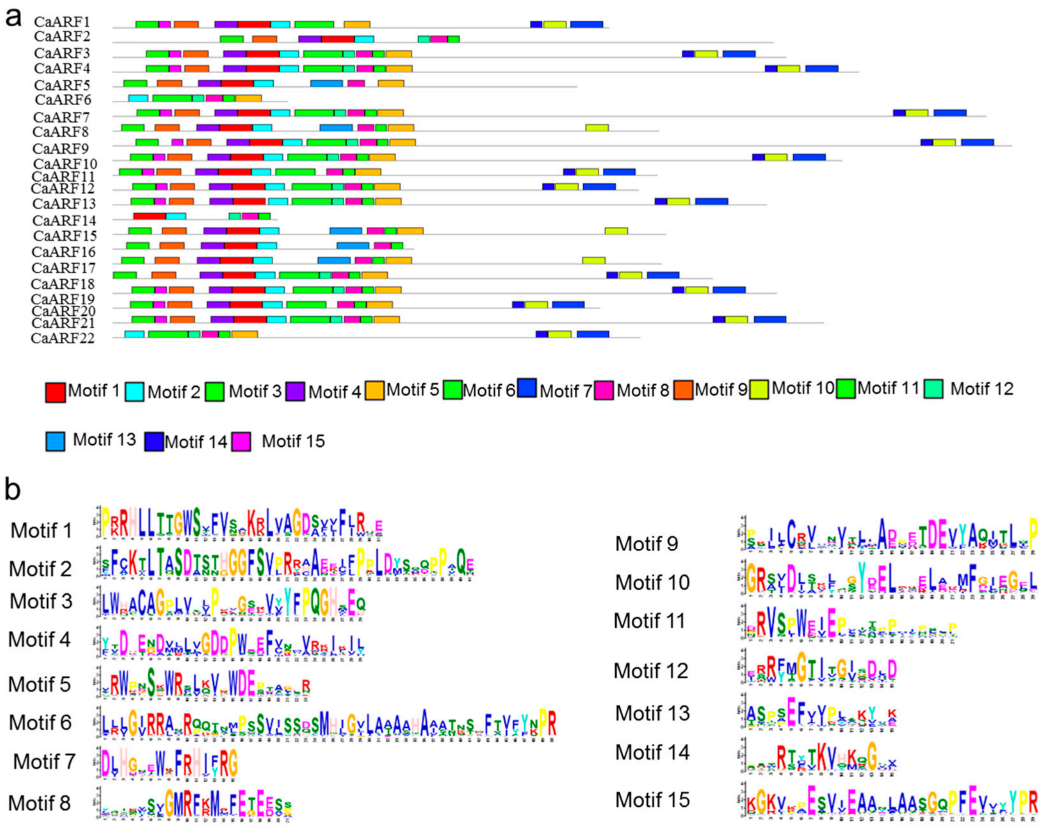

Figure S3. Conserved motif analysis of CaARF proteins. a. Conserved motif of CaARF proteins predicted by MEME tool. b. Conserved motif sequences of CaARF proteins.

a

```
CaARF5       5'--GGGCATGCAGGGAGCCAGGCA---3'
CaARF15      5'--TGGCATGCAGGGAGCCAGGCA---3'
CaARF8       5'--AGGCATACAGGGAGCCAGGCA---3'
CaARF17      5'--AGGCATACAGGGAGCCAGGCA---3'
                 :|||||:|||||||||||||||
AtmiRNA160   3'--ACCGUAUGUCCCUCGGUCCGU---5'
```

b

```
CaARF22      5'--AAGATCAGGCTGGCAGCTTGT---3'
CaARF13      5'--GAGATCAGGCTGGCAGCTTGT---3'
CaARF21      5'--GAGATCAGGCTGGCAGCTTGT---3'
                 :|||||| :||: ||||||||| :
AtmiRNA167   3'--AUCUAGUACGACCGUCGAACU---5'
```

Figure S4. Sequences of *AtmiRNA160* (a) and target *AtmiRNA167* (b) sites on *CaARF* mRNAs. The Watson-Crick base pairings to the *AtmiRNA160* and *AtmiRNA167* sequence are shown.

Table S1. List of primers used in the present study.

Gene name	Forward primer(5'to3')	Reverse primer(5'to3')
CaARF1 qRT	ACAAACCGAGATATATGGGCA CT	GCCTTTTGTTCTTCCCTGCG
CaARF2 qRT	TCTCTGCACAGTGATCAGTCA	GGGTATAGGGGAAGGGTGGT
CaARF3qRT	TACGGAGCTTTGGCGTTCAT	CACCACGCGGCATAGTATCT
CaARF4 qRT	AGATGGCCTGGTTCCAAGTG	CGCCCCCAAAAAGGTAGACT
CaARF5 qRT	CCCTTCATTCTCTGCCGTGT	CAACCTCCTTCTCCACGTCC
CaARF6 qRT	AACTTCTCACATTGGCAGTGCT C	GAGTTGCACGACGAATTCCC
CaARF7 qRT	TTGGGCCAATCCAGGATCAC	CCTCCCCGTGCAGTTATTGT
CaARF8 qRT	TCTGGCCGTTTAGGCACATT	CCGATTGGGTTTCCTGTCCA
CaARF9 qRT	AGCATACTGCCAACGGGAAT	GCTGCAACCCCTTCAAAAGAA
CaARF10 qRT	AGCCTGTCGTGACACCATTT	TGGTTTGGTATCAAGCTCAGTC
CaARF11 qRT	ACAAGCAGCTGATCACACGA	AGCTGAGCTGGATGCAACAT
CaARF12 qRT	TGCTTTCATCTTTCTCAGGCAA	AACACCGAGAAGAAGGTGCC
CaARF13 qRT	ATATATTTCGCGGACAAGCGG	CTGCTGCAGCTAGAAGACCA
CaARF14 qRT	ATTGGTTGCTGGCAATGCTT	ATGAATCCCAAGACGAGAAGAA G
CaARF15 qRT	TTCACCGTGGAGGCTTCTTC	GGTGGCCATCGAGAGGAAAA
CaARF16 qRT	CGCGGAGGGTATTCAGCTTT	ACGTGACGCCTTCACAAAGA
CaARF17 qRT	AACCCGTGTGCTCTATCACG	GTTGCGAGACCAGTGATCCA
CaARF18 qRT	TCAGGAGCTGATTGCCAAGG	AGCCCGTTTCTAGGTCTTGC
CaARF19 qRT	TCGGAACAAGAACTGGCTGG	TAGCATCATAGCCTTCTGCAACA
CaARF20 qRT	TCACATGCAAGGGGTAGCTG	GCCAAGGATGATCGCCCATA
CaARF21 qRT	GGTTTCTTGCAGTCCTCCGA	CCAACAAGCCTTCTAGCCCA
CaARF22 qRT	ATGTGGAAGGAAGGACAGCC	CAGTCTGGGGTCGAATTGCT
CaACTIN	GCCCCTTGTTTGTGACAATG	ACACGAAGCTCATTGTAGAA
CaARF2 qRT	GGTACCATGGCAACGATTTATC TCTG	GTCGACCAGAGCAATATCAAGA AGCA
CaARF10 qRT	GGTACCATGAAGGCACCATCA AACGG	GTCGACTTCAGTTCCACTGCTAG CTT
CaARF12 qRT	GGTACCATGGCAAACCTTTCTG GAAA	GTCGACTGCTTTCCCCTCATTGCT AA
